# Butterfly gliomas: to biopsy or to ablate – a longitudinal cohort study

**DOI:** 10.1007/s11060-026-05655-8

**Published:** 2026-06-05

**Authors:** Vratko Himic, Morgan Leigh Johnson, Anshul Ratnaparkhi, Roxanne C. Mayrand, Jay Chandar, Vaidya Govindarajan, Daniel C. Kreatsoulas, Arman Jahangiri, Zachary C. Gersey, Daniel M. Aaronson, Ricardo J. Komotar, Micheal E. Ivan, Ashish H. Shah

**Affiliations:** 1 Department of Neurological Surgery, University of Miami Miller School of Medicine, Miami, FL USA; 2 Department of Neurosurgery, Loma Linda University Medical Center, Loma Linda, CA USA; 3 Division of Neurological Surgery, Saint Louis University School of Medicine, St. Louis, MO USA

**Keywords:** Biopsy, Glioma, Butterfly, Laser, Outcomes

## Abstract

**Purpose:**

Butterfly gliomas are an aggressive subset of high-grade glioma characterized by bilateral hemispheric involvement. These patients often only receive biopsy followed by chemoradiation rather than surgical resection. Laser interstitial thermal therapy (LITT) provides a minimally invasive alternative that can improve survival.

**Methods:**

We retrospectively compare outcomes in biopsy alone versus LITT cohorts. Demographic characteristics, perioperative outcomes, and survival metrics were analyzed. Within the LITT cohort, procedural and volumetric analyses were performed to examine the relationship between extent of ablation (EOA), residual tumor burden (RTB).

**Results:**

Of 44 patients, 15 underwent biopsy only and 29 received LITT. LITT was associated with a longer median overall survival compared to biopsy (14.86 versus 4.93 months, ***p*** = **0.0489**), and median progression-free survival (4.67 versus 2.53 months, ***p*** = **0.0389**). Within the LITT cohort, larger preoperative tumor volumes were associated with lower EOA (r²=0.46, *p* **= 0.0002**) and a higher RTB (r^2^ = 0.90, ***p*** **= < 0.0001**). Longer ablation times correlated with a larger EOA (r^2^ = 0.21, *p* **= 0.0320**). Neither EOA nor RTB were associated with survival. Postoperative KPS improvement correlated with improved OS after multivariable Cox proportional hazards analysis (HR (death) = 0.93, 95%CI:0.87–0.99, ***p*** **= 0.026**), with a similar trend in PFS. Total operating room time was longer in the LITT group (median 4.0 vs. 2.57 h, ***p*** = **0.0034**), while time-to-chemoradiation, ICU stay, and hospital stay were comparable. Post-operative re-admissions and complications were not statistically different between groups.

**Conclusions:**

LITT is associated with improved survival compared with biopsy alone without increased perioperative morbidity. Reliance on volumetric thresholds alone may be insufficient to evaluate treatment adequacy in LITT, particularly in complex and heterogeneous tumors.

## Introduction

Butterfly gliomas represent a rare and particularly aggressive subset of glioma, characterized by bilateral hemispheric involvement and inclusion of the corpus callosum. These tumors account for approximately 3–14% of all glioblastomas (GBMs) and are associated with an exceptionally poor prognosis, with median survival typically ranging from 3.2 to 6.3 months [1, 2].

Historically, butterfly gliomas have been considered surgically inaccessible due to the deep-seated location and risk of bilateral neurological injury, leading to a predominant biopsy and chemoradiation treatment approach [3–7]. However, this approach has been increasingly questioned [8]. Several retrospective studies and meta-analyses have demonstrated that surgical resection, when safely achievable, provides survival benefits compared to biopsy alone [3, 9]. Despite these findings, the neurological morbidity associated with resection, including motor, speech, and cognitive deficits, remains a significant concern.

Laser interstitial thermal therapy (LITT) has emerged as a minimally invasive alternative for treating deep-seated or surgically inaccessible brain tumors [10–14]. This MRI-guided stereotactic ablation technique delivers controlled thermal energy to achieve cytoreduction while minimizing surgical access morbidity. Beyond cytoreduction, LITT induces disruption of the blood-brain barrier, releases damage-associated molecular patterns, and may enhance immune activation in the tumor microenvironment [15–18]. Of particular interest, multiple laser catheters can be used to ablate different corridors of the often-complex topography of a butterfly glioma since these tumors are typically large, aggressive with a non-spherical and widely heterogenous form.

While LITT has been investigated for both newly diagnosed and recurrent cases in surgically inaccessible locations [9, 19, 20], its specific role in butterfly glioma management is less clear. Recent studies have shown safety and feasibility in butterfly glioma specifically; however, these earlier studies are often limited by smaller initial experience sample sizes and lack of comparative data [21, 22].

Given the unique anatomical challenges of butterfly glioma, the lack of effective treatment options, the demonstrated limitations of biopsy (rather than cytoreductive management), and the evolving understanding of LITT’s therapeutic mechanisms, a systematic comparison of these treatment modalities is warranted. We present a retrospective cohort study comparing outcomes in patients with butterfly glioma following biopsy compared to a LITT cohort. We focus particularly on survival metrics, as well as the post-operative complications, re-admission and neurological function, in addition to length-of-stay metrics as well as the interaction of LITT-specific metrics such as extent of ablation (EOA) and residual tumor burden (RTB). We explore the extent to which traditional volumetric approaches are relevant in heterogenous and complex patient cohorts.

## Methods

### Patient selection

We performed a retrospective study of patients undergoing biopsy or LITT for butterfly glioma at our institution. Patients were included if they had radiographic evidence of a supratentorial tumor crossing the midline with clear bilateral hemispheric involvement, histopathologic confirmation of glioma, and underwent either stereotactic needle biopsy alone or stereotactic biopsy followed by LITT for this lesion (LITT occurs via the same biopsy tract in the same procedure).

### Ethical approval and declarations

This study received ethical approval by the Institutional Review Board at the University of Miami (IRB#20160437); the board decided that the retrospective nature of our study did not necessitate explicit informed patient consent and this requirement was waived.

### Biopsy procedure

All procedures were performed under general anesthesia. Each patient was positioned on the operating table according to the planned trajectory, with the head secured in the corresponding stereotactic system. Three-dimensional contrast-enhanced MRI scans were obtained one day prior to surgery, and uploaded into the stereotactic system, which was used to assist trajectory and entry point planning. Fiducial markers were applied based on the surgeon’s preference. Following coordinate registration, trajectory confirmation was performed using an intraoperative O-arm scan. A stereotactic robot-assisted system (ROSA ONE^®^ Brain, Zimmer Biomet, Warsaw, Indiana, USA) was used. In each case, several biopsy cores were obtained using the biopsy needle compatible with the respective system. Sampling sites included several points along the planned trajectory depending on perceived diagnostic requirements.

### LITT procedure

In addition to the above biopsy procedure, patients receiving LITT underwent the following. In our LITT procedures, the biopsy is taken first followed immediately by the insertion of the laser catheter. Pre-operatively, trajectories are plotted to exclude all critical structures including vessels, ventricles, and eloquent brain areas using ROSA. An ablation corridor is planned to maximize ablation extent whilst avoiding critical neurovascular structures. The Visualase System (Medtronic, Minneapolis, Minnesota, USA) was used to perform all LITT procedures. Following the biopsy, the laser catheter is introduced through the same tract to the target and secured on bone anchors. For patients undergoing bilateral LITT (two catheter tracts, common in large bilateral gliomas in our institution), this procedure is repeated for the second ablation tract. After bone fiducial removal and skin closure, the patients is transferred to MRI for the MR-guided portion of LITT. After intraoperative confirmation of laser placement via MRI, temperature limits are set around the planned ablation cavity to maintain safety of surrounding structures. After an initial test dose, the laser is activated, with parameters guided on a case-by-case basis by a neurosurgeon experienced in LITT. The thermal thresholds and effects are monitored using continuous MR thermometry using proton resonance frequency shift-based data from gradient echo pulse sequences. A pullback occurs when the catheter is retrieved to reduce the depth of ablation and create a corridor of ablation. Following LITT, the skin is closed and the patient is extubated and transferred to the neurosurgical intensive care unit. A postoperative MRI is attained overnight, and patients are typically discharged the next day.

### Data collection, outcomes, and metrics

Demographic and clinical data were extracted from the electronic medical records and included age at diagnosis, sex, presenting symptoms, Karnofsky performance status (KPS) (pre-operatively, post-operatively and at last-follow-up), type of procedure, length of hospital/ICU stays and length of surgery. Lesion characteristics included lesion location, recurrence status, histopathological diagnosis, isocitrate dehydrogenase (IDH) mutation status, and O-6-methylguanine-DNA methyltransferase (MGMT) promoter methylation status. Post-operative details extracted included immediate and postoperative complications within 30 days, new or worsening neurological deficits, and re-admission under the neurosurgical team, as well as time to initiation of chemoradiation. Available post-procedural oncologic treatment data were collected, including whether patients proceeded to chemoradiation and time from procedure to initiation of chemoradiation. Further LITT specific details included number of catheters (trajectories), the ablation time and number of pullbacks. Progression-free survival (PFS) was defined as the days from procedure to radiographic confirmation of disease progression. Overall survival (OS) was defined as the days from surgery to the date of death. Only patients with greater than 45 days follow-up were included in survival analyses to exclude patients with insufficient follow-up for outcome assessment. Patients were censored at last-known-follow-up for PFS and at their last date known to be alive for OS. Comparison survival analysis was done using the logrank (Mantel-Cox) test and displayed on Kaplan-Meier curves. Pre-operative MR images were acquired on POD − 1, and all post-operative images were taken on POD1.

### Volumetric analysis

All imaging data were processed using the Functional MRI of the Brain Software Library (FSL, version 6.0.0 [23]. Regions of interest (ROIs) were defined from pre-operative and post-operative anatomical scans with radiologically confirmed ablation. For pre-operative segmentation, contrast-enhancing (CE) tumor was delineated on post-contrast T1-weighted MPRAGE images. For post-operative segmentation, ablation cavities were delineated using both post-contrast T1-weighted images and T2-weighted fat-suppressed sequences to improve visualization of the ablation zone and surrounding changes. All ROIs were manually segmented slice-by-slice in 3D Slicer (version 4.11.20210226) using threshold-assisted manual delineation by a neuro-imaging researcher (RCM) and subsequently reviewed by a senior neurosurgeon in our team. Segmentation masks were exported and processed in FSL. Pre-operative CE masks and post-operative ablation masks were binarized, and voxel counts and volumetric measurements (mm³) were obtained using *fslmaths* and *fslstats* functions. For each patient, pre-operative tumor volume and post-operative ablation volume were calculated independently. Extent of ablation (EOA) was defined as$\:\:EOA=\:({V}_{ablation}/{V}_{pre})\:\times\:\:100$. An additional metric was calculated: residual tumor burden (RTB). RTB was calculated to account for the absolute amount of tumor remaining after ablation, integrating both preoperative tumor volume and extent of ablation: $\:{RTB=V}_{pre}\times\:(1-EOA/100)$. RTB was defined as a binary variable regarding whether there was any residual tumor present on the postoperative scan that was not ablated. An EOA of 100% or more would be classified as no RTB, and an EOA of less than 100% would be classified as RTB, analogous to the binary distinction of gross total resection in craniotomies.

### Statistical analysis

Continuous variables were expressed as mean ± standard deviation (SD) or median with interquartile range (IQR) depending on distribution. Comparisons involving small sample sizes were performed using Fisher’s exact test. Correlations between continuous variables were assessed using Spearman’s rank correlation coefficient. Survival outcomes were analyzed using Kaplan–Meier methods and compared using the log-rank (Mantel–Cox) test. Overall survival (OS) and progression-free survival (PFS) were further evaluated using Cox proportional hazards regression. To assess the relevance of lesion ablation extent, EOA and RTB were evaluated as measures of treatment adequacy, assessed as continuous measures and using multiple dichotomized thresholds informed by prior literature and cohort distribution. Given the limited sample size and number of events, multivariable Cox regression analyses were restricted to parsimonious multivariable models to minimize overfitting. Variables of clinical relevance, including residual tumor burden (RTB) and early postoperative change in functional status (ΔKPS), were selected a priori for inclusion in the final models. ΔKPS was defined as the difference between preoperative KPS and KPS at the first two-week postoperative follow-up. Additional exploratory Cox sensitivity models were performed including age, preoperative tumor volume, ΔKPS, and either EOA or RTB to assess whether volumetric variables retained an association with survival after adjustment for clinically relevant covariates. Statistical analyses were performed using GraphPad Prism (GraphPad Software, San Diego, CA). All statistical tests were two-sided, and a p-value < 0.05 was considered statistically significant.

## Results

### Demographics and clinical characteristics

A total of 44 patients with butterfly gliomas were included (mean age = 66.68, 55% female). Of these, 15 underwent biopsy only and 29 underwent LITT. No statistically significant differences were observed in measured baseline characteristics between the two cohorts, including their age, sex, KPS, recurrent status, pre-op deficit or any other pre-operative clinical features, including their volumes (Table 1). The cohort was predominantly IDH-wildtype glioblastoma, with three IDH-mutant tumors included. Apart from three patients (two with anaplastic astrocytoma and one with gliosarcoma), all patients had glioblastoma (GBM). Regarding the procedure itself, the operation time (recorded here as being the total time the patient spent in the operating room including anesthetic and post-operative tasks, not the length of the neurosurgical component itself) was longer in the LITT cohort than the biopsy cohort (median 4 h versus 2.57 h, *p* = 0.0034). The length of ICU and hospital stay were not statistically significantly different (Table 1).

### Post-operative course

Of the 15 patients undergoing biopsy alone, one patient experienced a postoperative complication (6.7%). This patient developed a sudden-onset intracerebral hemorrhage the night after the biopsy with new onset hemiparesis and dysphasia. Given the patients age and co-morbid status, and following rapid post-operative decline, the team and family opted for discharge to the hospice service. There was one complication in the LITT cohort, where the patient developed hydrocephalus and required admission for ventriculoperitoneal shunt (VPS) insertion. 2/15 (13.3%) biopsy and 3/29 (10.3%) LITT patients developed worsening/new neurological deficits (Table 1). In the LITT cohort, 3/29 patients had to be re-admitted within 30d, two of those for rapid progression of disease with confusion, seizures and worsening symptoms, the third was admitted for the aforementioned VPS. The KPS post-operatively and at last available follow-up was not statistically different. The time to the onset of chemoradiation was longer in the LITT cohort (median of 28.5d) compared to the biopsy cohort (17d), but this was not significant (*p* = 0.0643).

**Table 1:** Summary of pre-, intra- and post-operative details common to both biopsy and LITT cohorts

Total	*n* = 15	*n* = 29	*n* = 44		
Characteristics	Biopsy	LITT	Total	Odds ratio (95% CI)	*p* value
**Age (mean)**	66.57	66.74	66.68		0.9636
**Sex**					
Male	4	16	20	3.39 (0.93–11.18)	0.1114
Female	11	13	24		
**Pre-Op KPS (median)**	80	80	80		0.9757
**Pre-Op Deficit**	7	16	23	0.71 (0.19–2.43)	0.7520
**Recurrent**	1	6	7	0.27 (0.02–2.18)	0.3926
**Location**					
Frontal	6	12	18		0.0797
Parietal	6	16	22		
Splenium	3	1	4		
**Pre-operative volume (median)**	34.8	27.95	31.1		0.2883
**Diagnosis**					
Glioblastoma	13	28	41		0.1110
Anaplastic astrocytoma	2	0	2		
Gliosarcoma	0	1	1		
**IDH status**					
IDH wild-type	13	28	41	0.23 (0.02–2.21)	0.2643
IDH mutant	2	1	3		
**MGMT status**					
MGMT -	2	8	10		0.4132
MGMT +	3	8	11		
MGMT Not Reported	10	13	23		
**Operation time**,** hours (median)**	2.57	4.00	3.81		***0.0034***
**Length of ICU stay**,** hours**	25	22	22		0.1522
**Length of Hospital Stay**,** days**	3	2.09	2.14		0.4098
**New post-operative deficit**	2	3	5	1.33 (0.21–7.15)	0.9999
**Neurosurgical complication within 30d**	1	1	2		
**Diagnosis-related readmission**	0	3	3		
**Underwent chemo/radiotherapy**	13	23	36	1.130 (0.23–6.56)	> 0.9999
**Time to chemoradiation (days)**	17	28.5	24		0.0643
**Last follow-up (median**,** (IQR))**,** days**	117 (203)	114 (178)	116 (203)		0.9662
**Post-Op KPS**	70	70	70		0.9861
**KPS at Last Follow-Up**	70	60	65		0.6600

The only significant difference was in the total time spent in the operating room, where LITT was longer than biopsy only by about an hour and a half, accounting for the need to introduce the laser catheters, transfer the patient into the MRI suite and carry out the ablation. Statistical comparisons report either Fisher’s exact test for count contingency table analysis, or Mann-Whitney test for continuous variables.

KPS – Karnofsky Performance Status; CI – Confidence interval; IDH – Isocitrate dehydrogenase; MGMT - O-6-Methylguanine-DNA Methyltransferase; ICU – Intensive care unit

### LITT was associated with longer overall and progression-free survival

**Fig. 1:**
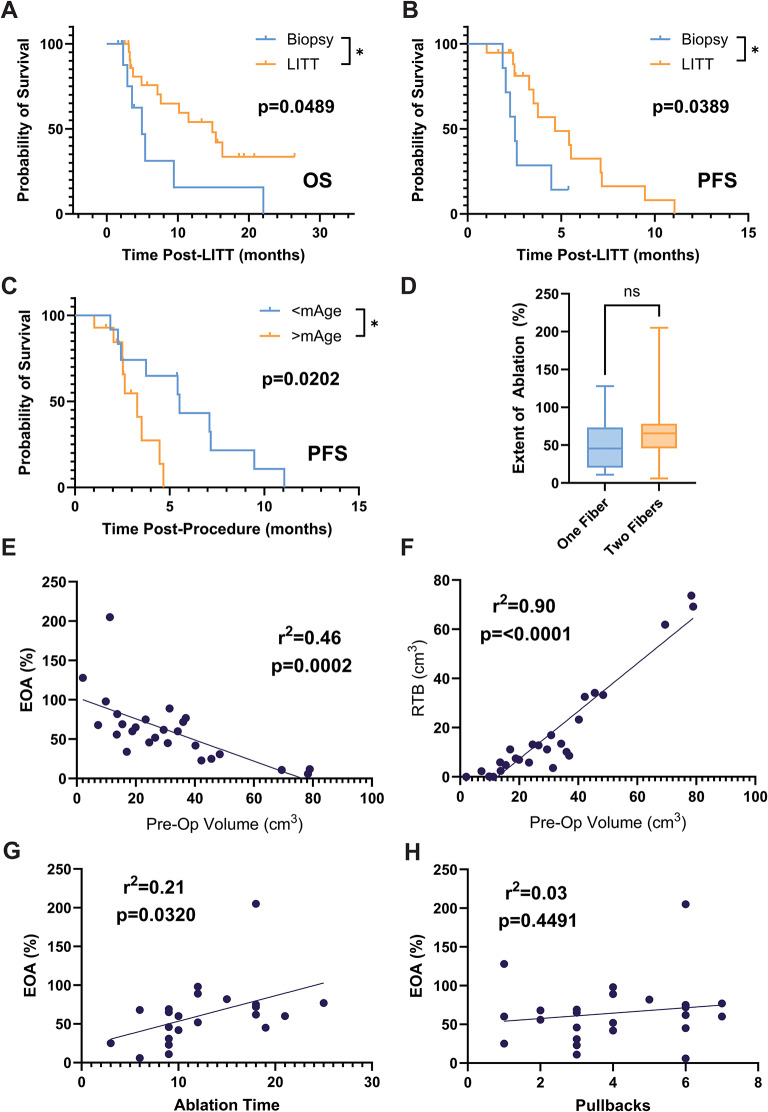
Post-operative patient outcomes in patients with butterfly glioma undergoing biopsy or LITT. **A-C** – Biopsy and LITT cohorts. **D-H –** LITT cohort only. **A –** Kaplan-Meier survival analysis showing that LITT prolongs median OS by almost 10 months (4.93 m vs. 14.86 m in the LITT cohort (*p* = 0.0489)). **B** - The median PFS is prolonged by 2 months (2.53 m vs. 4.67 m in the LITT cohort (*p* = 0.0389)). **C** – Younger patients (defined by the median cut-off of 68.1 years) (regardless of biopsy or LITT grouping), have a longer mPFS than older patients (5.52 m vs. 3.29 m, *p* = 0.0202). **D** – number of catheters (one versus two trajectories) does not affect EOA. **E-F** – pre-operative tumor volume inversely correlates with EOA (**E**) and positively correlates with RTB (**F**); in short, larger tumors achieved less ablation, as would be expected. **G** – ablation time demonstrates a mild positive correlation with EOA. **H** – there is no correlation number of pullbacks and EOA. EOA – Extent of ablation; RTB – Residual tumor burden

When comparing the outcomes in the LITT versus the biopsy-only cohort, LITT was associated with longer OS and PFS (Fig. 1a-b). In OS, the median OS (mOS) increased from 4.93 m in the biopsy to 14.86 m in the LITT cohort (**p** = **0.0489**). The median PFS (mPFS) increased from 2.53 m in the biopsy to 4.67 m in the LITT cohort (**p** = **0.0389**). In addition, patients who were younger than the median age of our overall butterfly glioma cohort of 68.1 years (regardless of biopsy or LITT grouping), had a longer mPFS than older patients (5.52 m versus 3.29 m, **p** = **0.0202**, Fig. 1c), with a similar, albeit non-significant trend, in mOS (11.51 m in younger, 7.13 m in older).

### Interaction of ablation characteristics and outcomes

Turning now specifically to the LITT cohort, we further analyzed the interaction of different features of LITT as well as volumetric analysis of the lesion and the ablation volumes, with descriptive statistics as follows: median pre-operative volume of 27.95cm^3^ (IQR:14.98–40.70); median ablation volume of 11.40cm^3^ (IQR:9.12–17.69); median EOA of 60% (IQR:33.25–75.50); median RTB of 10.66cm^3^ (IQR:4.52–25.61); median ablation time of 12 min (IQR:9–18), and a median of 4 pullbacks (IQR:1–6). Dual catheters were used in 58% of cases, the rest being single trajectory LITT. There was no difference in EOA when comparing single or dual trajectories (*p* = 0.2685, Fig. 1d).

As expected, we saw a large range of EOAs across the LITT cohort, representing the large spectrum of shapes, proximity to critical neurovascular structures as well as pre-operative volumes. Indeed, there was a significant inverse correlation between pre-operative volume and EOA (r^2^ = 0.46, *p* = 0.0002, Fig. 1e), and a positive correlation with RTB (r^2^ = 0.90, **p = < 0.0001**, Fig. 1f). Ablation time showed a mild positive correlation with EOA (r^2^ = 0.21, *p* = 0.0320, Fig. 1g), with no correlation number of pullbacks and EOA (r^2^ = 0.03, *p* = 0.4491, Fig. 1h).

**Fig. 2:**
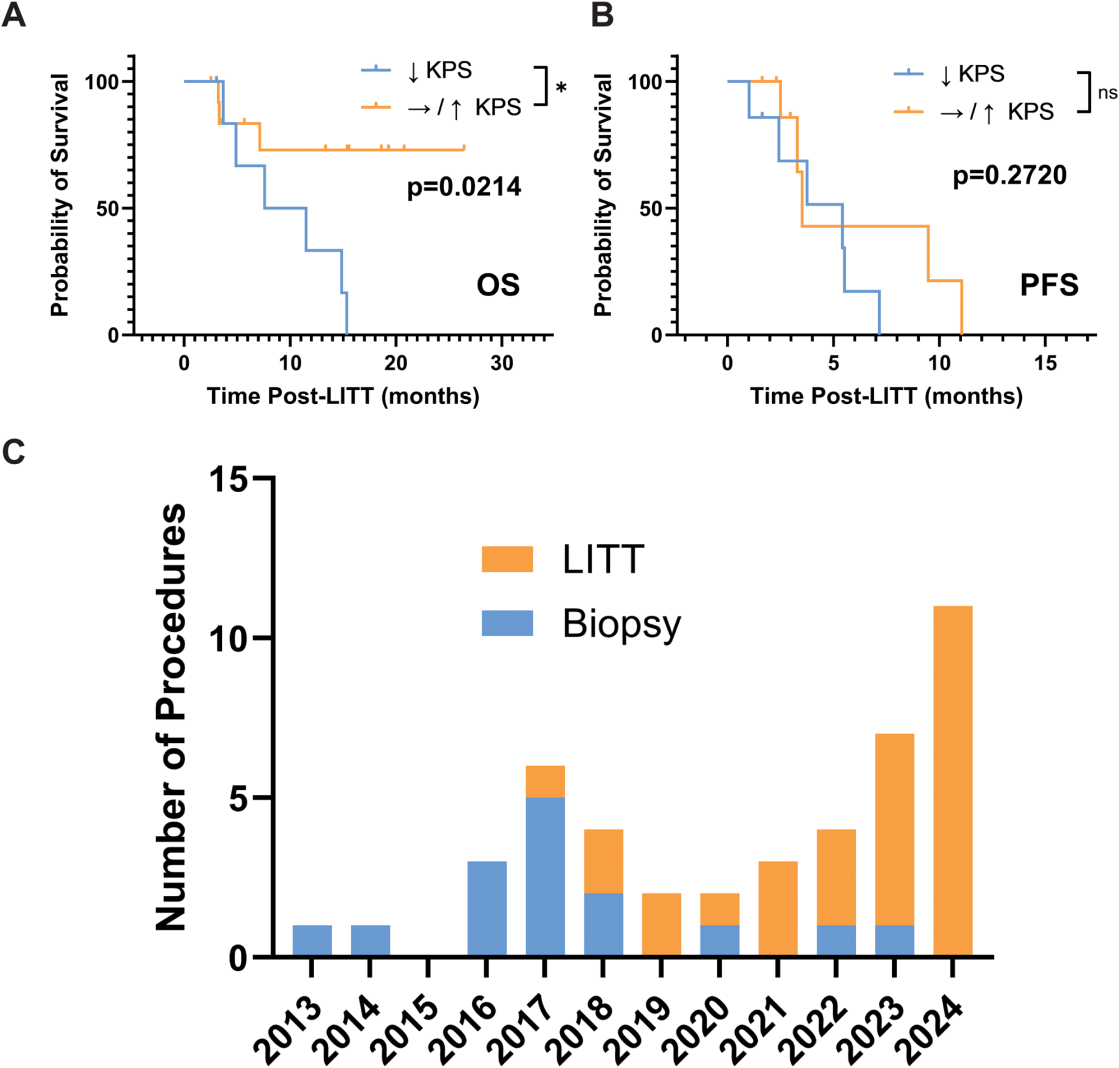
Change in KPS and the evolution of LITT over time. **A** – Kaplan-Meier analysis demonstrating that stable or improved KPS at the first post-operative follow-up visit (as compared to the pre-operative KPS) demonstrates an improved overall survival compared to those with a fall in KPS (*p* = 0.0214). The median OS for the reduced KPS group is 9.55 months, whilst the median OS was not reached in the stable/improved group due to censoring. **B** – Kaplan-Meier demonstrating the lack of statistically significantly difference in the PFS of patients based on their post-operative KPS, recapitulating the findings of the multivariable cox proportional hazards analysis. **C** – the evolution of biopsy and LITT over time shown as the number of each procedure performed from 2013 to 2024. NB - Although median postoperative KPS was lower at the cohort level, individual-level ΔKPS varied; therefore, ΔKPS analyses were performed using patient-level change rather than group medians

We further analyzed the RTB and the EOA and their potential contribution to LITT outcomes in the sub-group of butterfly glioma patients. However, across multiple analytical approaches, including threshold-based Kaplan–Meier analyses and univariate Cox proportional hazards modeling, neither EOA nor RTB demonstrated a consistent association with OS or PFS. However, using multivariable Cox proportional hazard analysis, early postoperative increase in KPS at the first follow up visit (two weeks post-operatively) was associated with improved OS (HR of death = 0.93, 95%CI:0.87–0.99, *p* = 0.026). This corresponds to an approximate 50% reduction in the hazard of death for a 10-point post-operative improvement in KPS. A similar but non-significant trend was observed for PFS (HR of progression = 0.95, 95%CI:0.87–1.03, *p* = 0.19). RTB was not associated with either OS (HR of death = 0.998 per cm³ of RTB, 95%CI:0.96–1.02, *p* = 0.87) or PFS (HR of progression = 0.98 per cm³ of RTB, 95%CI:0.94–1.01, *p* = 0.20). A visual representation of the association of KPS with survival is depicted in Fig. 2a-b, demonstrating improved OS in patients with stable or improved KPS compared to those with worsened KPS (**p** = **0.0214**). In exploratory multivariable Cox sensitivity models including age, preoperative tumor volume, KPS change, and either EOA or RTB, none of the variables were associated with OS or PFS. KPS change retained a protective direction across models, although the association was attenuated after additional adjustment, consistent with limited power and model instability.

### Evolution of practice over time

LITT has been available at our institution since 2013 (Fig. 2c). There was a statistically significant difference in the time distribution of these procedures with LITT occurring more recently post-2018 and biopsies more common pre-2018 (*p* = 0.0002), introducing potential treatment-era confounding due to the shift in procedural selection and represents a potential limitation.

## Discussion

In this article, we present the survival and safety longitudinal outcomes of patients with butterfly glioma treated with LITT compared to biopsy alone. In our cohort of 44 patients, LITT was demonstrated a statistically significant association with a nearly 10-month improvement in mOS and an 84% increase in mPFS relative to biopsy alone. While these survival outcomes are clinically meaningful, they were not reliably associated with extent of ablation, a well-established prognostic factor in non-butterfly glioma [24, 25], suggesting that alternative volumetric frameworks may be needed to evaluate cytoreductive efficacy in this tumor subtype.

**Fig. 3:**
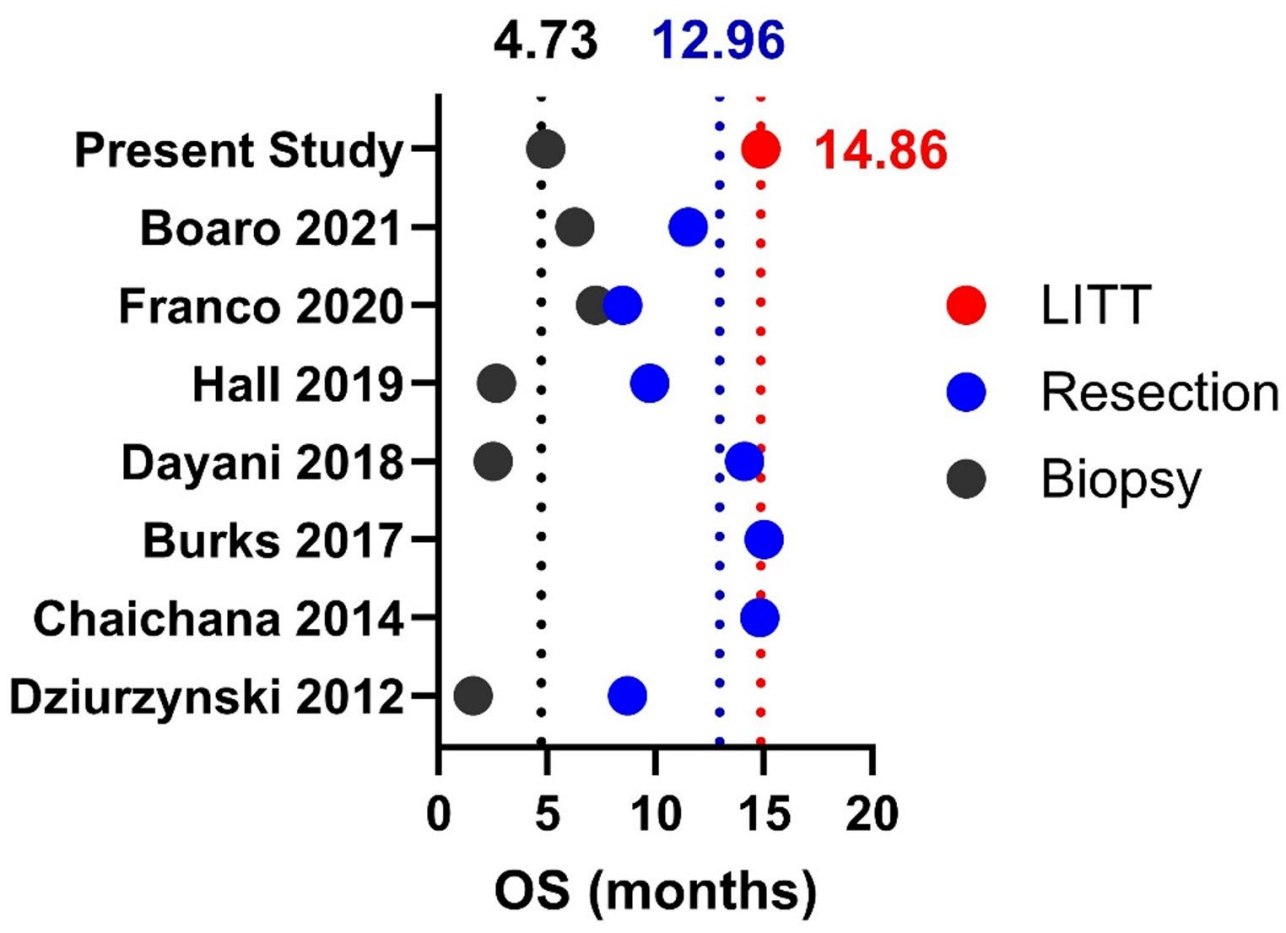
Median overall survival across published series of butterfly high-grade glioma stratified by treatment modality. Each point represents the reported median overall survival for an individual study (resection, biopsy, or LITT), where some studies reported one, some two modalities. To allow comparison across a heterogeneous literature, only median values are shown; measures of variance (e.g., 95% confidence intervals) were not included due to inconsistent reporting. Weighted means of reported median overall survival values were calculated for descriptive purposes only. Using this descriptive weighted mean method, the mean of median OS in biopsies and resection cohorts are demarcated by the black (4.73 months) and blue (12.96 months) dashed lines, respectively. Compared to the median OS in the present study using LITT of 14.86 months. (NB: This figure is intended as a descriptive visual summary rather than a formal meta-analysis. The visualization is descriptive and non-inferential and hypothesis-generating; no adjustment for patient selection, tumor burden, functional status, or treatment strategy was performed. No pooling or statistical comparisons were performed, as differences in patient traits, tumor characteristics, and treatment approaches limit direct comparability across the above studies)

The longer survival observed in the LITT cohort is both statistically significant and clinically substantial. Utilization of LITT was associated with an increase in median OS from 4.93 to 14.86 months and nearly doubled median PFS relative to biopsy alone. These findings extend prior work by Daggubati et al. which demonstrated a survival advantage for LITT over biopsy in a smaller cohort of butterfly glioma (OS:10.3 vs. 4.3 months, *p* = 0.035; PFS:5.5 vs. 2.8 months, *p* = 0.026) [21]. These prior early findings are now reinforced in a larger cohort. The present outcomes also compare favorably to open resection of butterfly glioma, where median OS has ranged from 7.0 to 15.0 months across institutional and national analyses [1, 4, 8, 26–29]. The median OS of 14.86 months in our LITT cohort meets these benchmarks, demonstrating the efficacy of LITT in prolonging OS in this patient cohort (Fig. 3). Notably, younger patients had significantly longer mPFS (5.52 vs. 3.29 months, *p* = 0.0202) with a similar non-significant trend in mOS (11.51 vs. 7.13 months), consistent with age as an established prognostic factor in butterfly glioma [3].

Importantly, the survival advantage conferred by LITT was not accompanied by increased morbidity. Complications were infrequent in both cohorts, with only one procedural complication occurring in each group: an intracranial hemorrhage following biopsy and hydrocephalus requiring ventriculoperitoneal shunt placement following LITT. New or worsening neurologic deficits were uncommon and did not differ significantly between groups (2/15 biopsy, 3/29 LITT). Of the three LITT readmissions within 30 days, two were attributable to rapid disease progression rather than procedural morbidity. These complication rates fall below those reported following open resection of butterfly glioma [30] and are consistent with those reported for LITT across broader neuro-oncological indications [31]. Time to chemoradiation was longer in the LITT cohort. However, this did not reach statistical significance, likely reflecting that while LITT is more involved than needle biopsy, it avoids the prolonged recovery associated with craniotomy [32–34].

**Fig. 4:**
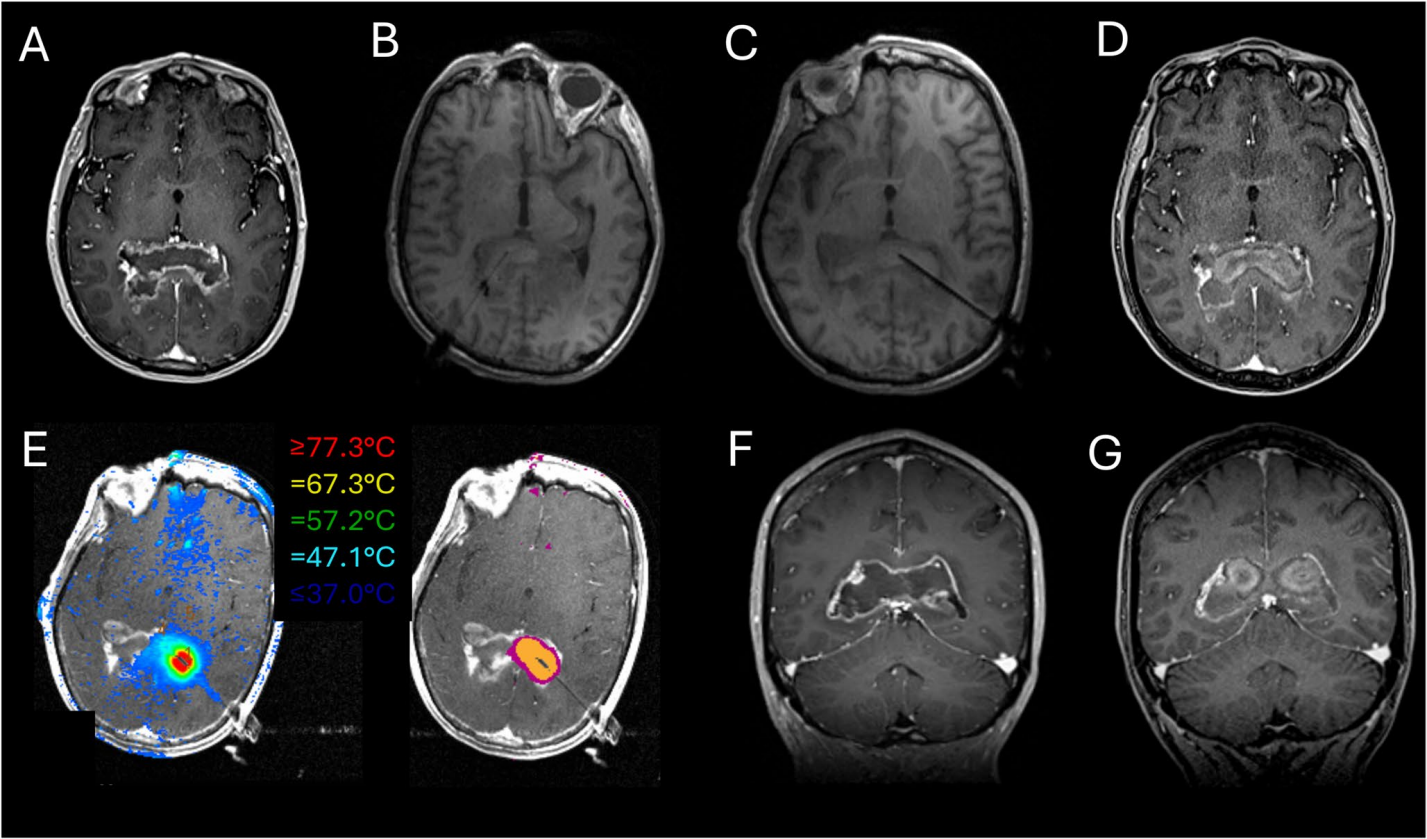
An example of a relatively favorable shape for successful large-volume ablation in LITT. A medium-large pre-operative volume (40.2cm^3^), with a subsequent ablation volume of 16.9cm^3^, EOA of 42.1% with an RTB of 23.30cm^3^. Even with this favorable shape showing two cylindrical paths on the axial scan (**A**) the coronal imaging reveals a much further inferior extension of the lesion (**F**). Therefore, even a dual catheter approach which achieves good axial control (**E**) struggles to achieve high EOAs due to the large pre-operative volume and the large extension inferiorly and medially along the ventricular system. This patient achieved a PFS of 3.52 months and was censored for OS (5.69 months). **A** – Axial pre-operative T1 with contrast MR image. **B** – Right and **C** – Left catheter approach intra-operatively. **D** – Post-operative axial T1 plus contrast image showing the ablation cavity. **E** – Intra-operative ablation heatmap (left panel) and ablation extent (right panel). **F** – Pre-operative coronal T1 with contrast MR image. **G** – Post-operative T1 with contrast image. All post-operative images taken on POD1

**Fig. 5:**
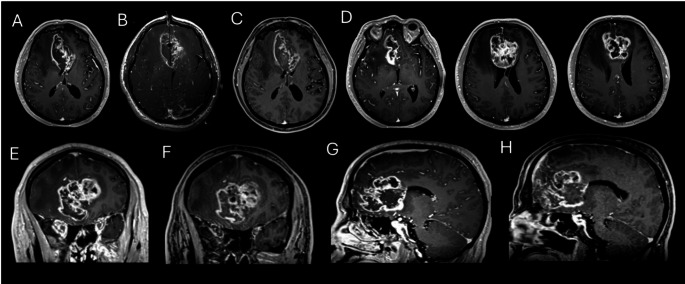
An example of an unfavorable shape for successful large-volume ablation in LITT. High pre-operative volume (69.4cm^3^), with a low subsequent ablation volume (7.50cm^3^) and a low EOA (11%) with a high RTB of 61.87cm^3^. The multiple loculations, closeness to heatsinks such as ventricles, dural folds and vasculature and the lack of an even, cylindrical structure all contribute to the overall EOA. Nevertheless, this patient achieved an OS of 19.3 months (censored) and a PFS of 11.05 months, suggesting that it is not as clear cut as smaller EOA causing worse survival in this aggressive subtype of tumors. **A** – Axial pre-operative T1 with contrast MR image. **B** – Laser catheter approach intra-operatively. **C** – Post-ablation scan showing ablation cavity enveloped by residual tumor. **D** – Further pre-operative axial T1 plus contrast image showing the large extent of the lesion. **E** – Pre- and **F** – Post-operative coronal T1 with contrast MR image. **G** – Pre- and **H** – Post-operative sagittal T1 with contrast MR image. All post-operative images taken on POD1

The relatively heterogeneous and low EOA observed in this cohort likely reflects two constraints inherent to butterfly glioma: shape and size. These tumors expand bilaterally from a midline epicenter along the corpus callosum, often producing an irregular mass that the LITT catheter, which ablates along a single conical trajectory, does not readily conform to. By way of example, compare the two different example cases from our LITT cohort in Figs. 4 and 5, and note the relatively LITT-friendly ablation corridor in the axial planes of Fig. 4, and the relatively LITT-unfriendly shape of the glioma in Fig. 5. Pre-operative volume was also significantly inversely correlated with EOA (r²=0.46, *p* = 0.0002), consistent with prior literature [11] describing that larger tumors receive proportionally less complete ablation. Accordingly, despite 70% being a well-defined prognostic threshold in non-butterfly glioma [12], the majority of our butterfly ablations did not meet this threshold.

We postulated that RTB might be a more prudent metric than EOA as even though a clinically meaningful volume of tumor may be ablated, the proportional EOA may still appear low: tumors with similar EOA can harbor vastly different absolute volumes of residual disease depending on preoperative size, and it is this residual disease burden that adjuvant therapy must ultimately address. This principle is established in the open resection literature, which has demonstrated that residual tumor volume may be more tightly associated with survival than extent of resection [35, 36]. However, attempts at stratification and correlation with either EOA or RTB did not demonstrate consistent associations with survival, although larger studies are needed to determine whether these metrics have prognostic value in anatomically complex butterfly gliomas.

It is therefore possible that it is not wise to generalize findings from the open resection literature to anatomically complex or highly variable tumors undergoing LITT. The wide range of tumor volumes, EOA and RTB values in this cohort likely limits the ability of purely volumetric methods to capture treatment impact. Indeed, consider the example case in Fig. 5, where with a very large pre-operative volume of 69.4cm^3^ and only 11% EOA with 61.87cm^3^ RTB, the patient achieved an OS of 19.3 months (censored) and a PFS of 11.05 months, suggesting that it is not as clear cut as ‘*smaller EOA leading to worse survival*’ in this aggressive tumor type.

By contrast, early postoperative change in KPS emerged as the only variable that demonstrated association with OS, suggesting that global patient response may better reflect underlying disease trajectory than volumetric measurements. One possible explanation is that the therapeutic effect of LITT extends beyond pure direct cytoreduction, potentially influencing the tumor microenvironment and enhancing responsiveness to subsequent therapies [17].

### Limitations

This study has several limitations. Primarily, butterfly glioma is a rare tumor subtype, and while this represents one of the largest cohorts reported to date, the sample size remains small, limiting statistical power for volumetric subgroup analyses including the RTB stratification. Furthermore, as a single-center retrospective analysis performed at a tertiary referral center, it is subject to selection bias and reduced generalizability. Some patients will have differences in family support, personal values, and health status that cannot be accurately and fully captured by retrospective chart review, and this may cause differences between groups. In this way, baseline variables may not capture differences in tumor eloquence, frailty, family support, goals of care, or other treatment-selection factors.

Because LITT was disproportionately performed later in the study period, temporal changes in neuro-oncological care, imaging surveillance, supportive care, and adjuvant treatment delivery including steroid exposure may have contributed to the observed survival differences. Whilst post-operative chemoradiation or time to chemotherapy were not significantly different between the two groups, more detailed chemoradiation completion, steroid exposure, and salvage-treatment data were not uniformly available because several patients were lost to follow-up, completed therapy at outside institutions, or declined further treatment. As such, differences in downstream oncologic therapy remain a potential confounder. Additionally, changes in KPS should be interpreted as an early clinical prognostic marker rather than treatment effects.

Additionally, due to the low numbers we could not effectively control for the effects of MGMT methylation or IDH mutant status, which are known to be contributory to survival, nor could we carry out even more comprehensive multi-variable adjustments given the risks of overfitting and multicollinearity. Given the limited number of events, the number of variables included in multivariable models was restricted to maintain an appropriate events-per-variable ratio. Although expanded Cox sensitivity models were performed, these analyses were limited by a low events-per-variable ratio and evidence of multicollinearity, particularly when preoperative volume was modeled alongside RTB, which is mathematically derived from volume and EOA. Therefore, these models were interpreted as exploratory and were not used to support definitive adjusted conclusions. We encourage and welcome future multi-institutional collaborations to enable more definitive adjusted analyses.

On a positive note, compared to prior studies that often deploy ellipsoid volumetric formulas which oversimplify and do not take into account the complex shape of butterfly gliomas, we have deployed a novel approach which we found to define EOA and pre/post-operative volumes much more accurately using 3D mask segmentation software.

## Conclusion

In this single-center retrospective cohort of patients with butterfly glioma, LITT was associated with longer overall and progression-free survival compared with biopsy alone, without a clear increase in perioperative morbidity. Despite these favorable outcomes, survival did not demonstrate an association with EOA or RTB, suggesting that traditional proportional metrics may inadequately capture LITT efficacy in this uniquely infiltrative tumor subtype.

## Data Availability

Data is available by contacting the corresponding author.

## References

[1] Dziurzynski K, Blas-Boria D, Suki D et al (2012) Butterfly glioblastomas: a retrospective review and qualitative assessment of outcomes. J Neurooncol 109:555–563. 10.1007/s11060-012-0926-0 22806339 10.1007/s11060-012-0926-0 PMC3992290

[2] Sinha S, Avnon A, Perera A et al (2023) Butterfly gliomas: a time for stratified management? Neurosurg Rev 46:223. 10.1007/s10143-023-02126-w 37665387 10.1007/s10143-023-02126-w PMC10477135

[3] Laviv Y, Kasper EM (2025) Butterfly glioblastoma: trends in therapeutic modalities, extent of resection and survival in the temozolomide era. a SEER-based study. Neurosurg Rev 48:406. 10.1007/s10143-025-03558-2 40338381 10.1007/s10143-025-03558-2 PMC12062142

[4] Burks JD, Bonney PA, Conner AK et al (2017) A method for safely resecting anterior butterfly gliomas: the surgical anatomy of the default mode network and the relevance of its preservation. J Neurosurg 126:1795–1811. 10.3171/2016.5.JNS153006 27636183 10.3171/2016.5.JNS153006 PMC9473322

[5] Chaichana KL, Jusue-Torres I, Lemos AM et al (2014) The butterfly effect on glioblastoma: is volumetric extent of resection more effective than biopsy for these tumors? J Neurooncol 120:625–634. 10.1007/s11060-014-1597-9 25193022 10.1007/s11060-014-1597-9 PMC4313925

[6] Balaña C, Capellades J, Teixidor P et al (2007) Clinical course of high-grade glioma patients with a biopsy-only surgical approach: a need for individualised treatment. Clin Transl Oncol 9:797–803. 10.1007/s12094-007-0142-0 18158984 10.1007/s12094-007-0142-0

[7] Parsa AT, Wachhorst S, Lamborn KR et al (2005) Prognostic significance of intracranial dissemination of glioblastoma multiforme in adults. J Neurosurg 102:622–628. 10.3171/jns.2005.102.4.0622 15871503 10.3171/jns.2005.102.4.0622

[8] Dayani F, Young JS, Bonte A et al (2018) Safety and outcomes of resection of butterfly glioblastoma. Neurosurg Focus 44:E4. 10.3171/2018.3.FOCUS1857 29852771 10.3171/2018.3.FOCUS1857

[9] Chojak R, Koźba-Gosztyła M, Słychan K et al (2021) Impact of surgical resection of butterfly glioblastoma on survival: a meta-analysis based on comparative studies. Sci Rep 11:13934. 10.1038/s41598-021-93441-z 34230597 10.1038/s41598-021-93441-z PMC8260698

[10] Shah AH, Burks JD, Buttrick SS et al (2019) Laser Interstitial Thermal Therapy as a Primary Treatment for Deep Inaccessible Gliomas. Neurosurgery 84:768–777. 10.1093/neuros/nyy238 29873756 10.1093/neuros/nyy238

[11] Shah AH, Semonche A, Eichberg DG et al (2020) The Role of Laser Interstitial Thermal Therapy in Surgical Neuro-Oncology: Series of 100 Consecutive Patients. Neurosurgery 87:266–275. 10.1093/neuros/nyz424 31742351 10.1093/neuros/nyz424

[12] Di L, Wang CP, Shah AH et al (2021) A Cohort Study on Prognostic Factors for Laser Interstitial Thermal Therapy Success in Newly Diagnosed Glioblastoma. Neurosurgery 89:496–503. 10.1093/neuros/nyab193 34156076 10.1093/neuros/nyab193 PMC8364818

[13] Knott MV, Himic V, Khalafallah AM et al (2026) Laser interstitial thermal therapy for intracranial dural-based lesions: A single center case series of 20 patients. Clin Neurol Neurosurg 265:109384. 10.1016/j.clineuro.2026.109384 41812351 10.1016/j.clineuro.2026.109384

[14] Shah KH, Khalafallah AM, Knott MV et al (2026) The safety and efficacy of laser interstitial thermal therapy for newly diagnosed deep-seated low-grade glioma: a pilot study comparing outcomes with a surgical cohort. Neurosurgery. 10.1227/neu.0000000000003951. 10.1227/neu.0000000000003951 41642008

[15] Leuthardt EC, Duan C, Kim MJ et al (2016) Hyperthermic Laser Ablation of Recurrent Glioblastoma Leads to Temporary Disruption of the Peritumoral Blood Brain Barrier. PLoS ONE 11:e0148613. 10.1371/journal.pone.0148613 26910903 10.1371/journal.pone.0148613 PMC4766093

[16] Himic V, Ramsoomair CK, Soldozy S et al (2025) The Future of Clinical Trials in Surgical Neuro-Oncology Is Bright. Brain Sci 15:1274. 10.3390/brainsci15121274 41440070 10.3390/brainsci15121274 PMC12730250

[17] Vargas LO, Himic V, Otaner F et al (2025) Modulating the glioma microenvironment with laser interstitial thermal therapy: mechanisms and therapeutic implications. J Neurooncol 176:99. 10.1007/s11060-025-05305-5 41307758 10.1007/s11060-025-05305-5 PMC12660436

[18] Chandar JS, Bhatia S, Ingle S et al Laser interstitial thermal therapy induces robust local immune response for newly diagnosed glioblastoma with long-term survival and disease control. J Immunother 46:351–354. 10.1097/CJI.0000000000000485. 10.1097/CJI.0000000000000485 PMC10591996 37727953

[19] Avecillas-Chasin JM, Atik A, Mohammadi AM, Barnett GH (2020) Laser thermal therapy in the management of high-grade gliomas. Int J Hyperth 37:44–52. 10.1080/02656736.2020.1767807 10.1080/02656736.2020.1767807 32672121

[20] Rangwala HS, Shafique MA, Mustafa MS et al (2024) Evaluating efficacy and safety of laser interstitial thermal therapy in patients with newly diagnosed and recurrent glioblastoma: a systematic review and meta-analysis. Neurosurg Rev 47:846. 10.1007/s10143-024-03077-6 39528836 10.1007/s10143-024-03077-6

[21] Daggubati LC, Ramos-Fresnedo A, Merenzon MA et al (2023) Bilateral Laser Interstitial Thermal Therapy for Butterfly Gliomas Compared With Needle Biopsy: A Preliminary Survival Study. Oper Neurosurg (Hagerstown) 25:435–440. 10.1227/ons.0000000000000850 10.1227/ons.0000000000000850 37846139

[22] Jamshidi AM, Eichberg DG, Komotar RJ, Ivan M (2020) Safety Analysis of Bilateral Laser Interstitial Thermal Therapy for Treatment of Butterfly Glioma. World Neurosurg 144:e156–e163. 10.1016/j.wneu.2020.08.053 32795682 10.1016/j.wneu.2020.08.053

[23] Jenkinson M, Beckmann CF, Behrens TEJ et al (2012) FSL Neuroimage 62:782–790. 10.1016/j.neuroimage.2011.09.015 21979382 10.1016/j.neuroimage.2011.09.015

[24] Gurses ME, Lu VM, Gecici NN et al (2024) Laser interstitial thermal therapy in neurosurgery: a single-surgeon experience of 313 patients. J Neurosurg 141:1281–1291. 10.3171/2024.3.JNS245 38820611 10.3171/2024.3.JNS245

[25] Mohammadi AM, Hawasli AH, Rodriguez A et al (2014) The role of laser interstitial thermal therapy in enhancing progression-free survival of difficult-to-access high-grade gliomas: a multicenter study. Cancer Med 3:971–979. 10.1002/cam4.266 24810945 10.1002/cam4.266 PMC4303165

[26] Chaichana KL, Jusue-Torres I, Navarro-Ramirez R et al (2014) Establishing percent resection and residual volume thresholds affecting survival and recurrence for patients with newly diagnosed intracranial glioblastoma. Neuro Oncol 16:113–122. 10.1093/neuonc/not137 24285550 10.1093/neuonc/not137 PMC3870832

[27] Hall BJ, Maleyko I, Brodbelt A et al (2019) The utility of surgery in butterfly glioblastoma: A case-control study. J Clin Oncol 37:e13529–e13529. 10.1200/JCO.2019.37.15_suppl.e13529

[28] Barritault M, Picart T, Poncet D et al (2020) Avoiding New Biopsies by Identification of IDH1 and TERT Promoter Mutation in Nondiagnostic Biopsies From Glioma Patients. Neurosurgery 87:E513–E519. 10.1093/neuros/nyaa025 32107549 10.1093/neuros/nyaa025

[29] Boaro A, Kavouridis VK, Siddi F et al (2021) Improved outcomes associated with maximal extent of resection for butterfly glioblastoma: insights from institutional and national data. Acta Neurochir (Wien) 163:1883–1894. 10.1007/s00701-021-04844-w 33871698 10.1007/s00701-021-04844-w

[30] Chawla S, Kavouridis VK, Boaro A et al (2022) Surgery vs. biopsy in the treatment of butterfly glioblastoma: a systematic review and meta-analysis. Cancers (Basel) 14. 10.3390/cancers14020314. 10.3390/cancers14020314 PMC8773472 35053478

[31] Alkazemi M, Lo YT, Hussein H et al (2023) Laser Interstitial Thermal Therapy for the Treatment of Primary and Metastatic Brain Tumors: A Systematic Review and Meta-Analysis. World Neurosurg 171:e654–e671. 10.1016/j.wneu.2022.12.079 36549438 10.1016/j.wneu.2022.12.079

[32] Buszek SM, Al Feghali KA, Elhalawani H et al (2020) Optimal Timing of Radiotherapy Following Gross Total or Subtotal Resection of Glioblastoma: A Real-World Assessment using the National Cancer Database. Sci Rep 10:4926. 10.1038/s41598-020-61701-z 32188907 10.1038/s41598-020-61701-z PMC7080722

[33] Pandey A, Chandla A, Mekonnen M et al (2024) Safety and efficacy of laser interstitial thermal therapy as upfront therapy in primary glioblastoma and idh-mutant astrocytoma: a meta-analysis. Cancers (Basel) 16. 10.3390/cancers16112131. 10.3390/cancers16112131 PMC11171930 38893250

[34] McGinley C, Himic V, Knott M et al (2026) Barriers to expeditious post-operative chemotherapy for patients with glioblastoma. Clin Neurol Neurosurg 264:109352. 10.1016/j.clineuro.2026.109352 41722183 10.1016/j.clineuro.2026.109352

[35] Grabowski MM, Recinos PF, Nowacki AS et al (2014) Residual tumor volume versus extent of resection: predictors of survival after surgery for glioblastoma. J Neurosurg 121:1115–1123. 10.3171/2014.7.JNS132449 25192475 10.3171/2014.7.JNS132449

[36] Gerritsen JKW, Zwarthoed RH, Kilgallon JL et al (2023) Impact of maximal extent of resection on postoperative deficits, patient functioning, and survival within clinically important glioblastoma subgroups. Neuro Oncol 25:958–972. 10.1093/neuonc/noac255 36420703 10.1093/neuonc/noac255 PMC10158118

